# Influence of Moisture Content on Electromagnetic Response of Concrete Studied Using a Homemade Apparatus

**DOI:** 10.3390/s19214637

**Published:** 2019-10-25

**Authors:** Zhe Li, Zuquan Jin, Shuangshuang Shao, Tiejun Zhao, Penggang Wang

**Affiliations:** 1 School of Civil Engineering, Qingdao University of Technology, Qingdao 266033, China; lizhequt@163.com (Z.L.); KY1718824180@163.com (S.S.); ztjgp@263.net (T.Z.); wangpenggang007@163.com (P.W.); 2 Cooperative Innovation Center of Engineering Construction and Safety in Shandong Blue Economic Zone, Qingdao 266033, China; 3 School of Mathematics, Computer Science and Engineering, City, University of London, London EC1V0HB, UK

**Keywords:** electromagnetic response, Hall effect, relative humidity, carbonation, water absorption, correction factor, concrete

## Abstract

In this study, we examined the influence of moisture content on the electromagnetic response of concrete. A novel homemade electromagnetic monitoring apparatus was developed and used to evaluate the Hall effect voltage at both ends of concrete based on our previous study of the Hall effect. We used four different concrete mix water/binder ratios: 0.30, 0.28, 0.26, and 0.24, and three conditions (relative humidity, carbonation, and water absorption) were examined in this experiment. The results show that the moisture content inside concrete influences the relative permeability of concrete. The variation in the Hall effect voltage is more influenced by carbonation than changes in relative humidity; water absorption increases the Hall effect voltage the least amongst the other examined factors. According to the experiment, a calibration system was established, and the relevant correction factors are provided.

## 1. Introduction

Reinforced concrete is widely used in the construction of most facilities due to its low cost and the availability of its constituent materials [1,2,3]. Reinforced concrete structures have the potential to be durable and capable of withstanding a variety of harsh environmental conditions [4,5]. However, a major cause of the durability problems experienced by reinforced concrete structures is steel bar corrosion, especially in marine environments. Steel corrosion not only reduces the cross-sectional area of steel bars but also leads to the dilation and cracking of concrete coverings. Consequently, the service life and safety of reinforced concrete structures decreases [6,7]. Reinforcements are placed inside concrete so the corrosion level of reinforced bars cannot be observed by the naked eye. Increasing numbers of non-destructive monitoring technologies are emerging, among which non-contact monitoring technology has become a significant method for detecting displacement, cracks, deformation, as well as corrosion degree inside the concrete or on the concrete surface. To evaluate and monitor the corrosion damage level of reinforced bars, the development of corrosion monitoring apparatus has become a research focus. With the development of electromagnetic theory, various types of innovative monitoring apparatus have been developed based on the many different magnetic properties of steel and concrete. Hong et al. [8] proposed long-term ground penetrating radar (GPR) to monitor corrosion behavior inside concrete. Zhang [9] reported a non-destructive test (NDT) of steel corrosion in reinforced concrete bridges using a micro-magnetic sensor. Rumiche [10] assessed the effect of microstructure inside concrete on the magnetic behavior of structural carbon steel using an electromagnetic sensor. Mao [11] reported the device-monitored corrosion process of reinforced concrete using BOTDA and FBG sensors. Gotoh [12] improved an electromagnetic non-destructive device to detect rust regions in steel in 2005. Yasri [13] highlighted a radiofrequency sensing method that is capable of monitoring uniform and localized corrosion. Sharma [14] combined several propagation techniques for monitoring invisible corrosion in concrete. Zhang and Liu [15,16] introduced a corrosion evaluation technique for reinforced concrete structures using Hall effect magnetic sensors. In a previous study, we developed two different versions of electromagnetic monitoring apparatuses to monitor steel bar corrosion [17,18].

Although many researchers have focused on corrosion monitoring apparatuses, numerical research discovered the significant influence of moisture content on the permittivity of concrete, which describes the reflection state when an electromagnetic wave is transmitted to a concrete surface. Despite these novel monitoring and detecting apparatuses, the relative permeability of dry concrete is assumed to be one, as it is a weakly magnetic medium; however, dry concrete rarely occurs in practice due to the existence of pore solutions inside the concrete [19,20,21]. Water is also a common kind of weak magnetic medium that mainly exists in concrete in the form of a pore solution; as a result, different moisture amounts inside concrete may affect its relative permeability. Zhang [15] also reported this limitation, which may further affect the monitoring signals acquired by the monitoring apparatus mentioned above. However, few studies have examined the electromagnetic response of concrete. To acquire accurate monitoring signals using an electromagnetic monitoring apparatus, the factors that may influence signals need to be calibrated. Considering moisture is the most significant factor influencing the relative permeability of concrete, relative humidity and water absorption are regarded as influencing factors. The carbonation effect is considered a significant factor influencing the durability of concrete. Although concrete undergoes a self-healing process, it can be almost ignored compared with carbonation-triggered corrosion, especially in marine environments [22,23]. Carbonation has always been reported to decrease chloride ion binding ability with concrete, which reaches a maximum on the concrete surface [24,25,26]. Chang [27,28] reported on carbonation-induced water release in a whole specimen, which has attracted attention worldwide. To uncover the relationship between concrete moisture and the effect of carbonation, Possan [29] reported a case study of CO_2_ uptake potential due to concrete carbonation. Based on previous research, the influence of carbonation on the accuracy of the monitoring signals of electromagnetic monitoring apparatus is still unknown, so requires further research.

The purpose of this investigation was to determine the influence of the concrete micro-environment and its water/binder (w/b) ratio on its electromagnetic response. Therefore, the relative permeability of concrete with four different w/b ratios (0.3, 0.28, 0.26, and 0.24) and effects of relative humidity, carbonation exposure, and water absorption were investigated. A novel homemade electromagnetic monitoring apparatus (EMMA) reported by Li and Jin [18] was used to detect the Hall effect voltage of concrete, which determines the relative permeability of concrete.

## 2. Electromagnetic Mechanics

### 2.1. Electromagnetic Transmission Mechanics

The transmission speed of an electromagnetic wave is defined in Equation (1). Equation (1) shows that two key parameters, μ and ε, determine the transmission speed v in media. As introduced above, it is uncertain whether moisture content influences the relative permeability of concrete media. When an electromagnetic wave enters a target objective with obvious differences in relative permittivity in the process of transmission in the medium, reflection and transmission may occur. The energy distribution coefficient is defined in Equation (2). If the relative permeability of concrete is assumed to be one, the energy distribution coefficient will be approximately as shown in Equation (3), which indicates the unreasonable point that the influence of relative permeability on electromagnetic transmission cannot be taken into consideration [30,31].

(1) $$v=\frac{1}{\sqrt{\mu \varepsilon }}$$

(2) $$\gamma =\frac{{\left(\sqrt{\varepsilon_{2}\mu_{2}}-\sqrt{\varepsilon_{1}\mu_{1}}\right)}^{2}}{{\left(\sqrt{\varepsilon_{2}\mu_{2}}+\sqrt{\varepsilon_{1}\mu_{1}}\right)}^{2}}$$

(3) $$\gamma =\frac{{\left(\sqrt{\varepsilon_{2}}-\sqrt{\varepsilon_{1}}\right)}^{2}}{{\left(\sqrt{\varepsilon_{2}}+\sqrt{\varepsilon_{1}}\right)}^{2}}$$

where $\mu_{1}$ and $\mu_{2}$ are the relative permeability of two media, $\varepsilon_{1}$ and $\varepsilon_{2}$ are the permittivity of two media, and γ is reflection coefficient.

### 2.2. Mechanics of Moisture Content Influencing Relative Permeability

As a non-magnetic material, the relative permeability of concrete has always been assumed to be one. However, the relative permittivity can be expressed in the form of relative complex permittivity, which is shown in Equation (4). The relative complex permittivity is a dimensionless complex number. The real part represents the storage coefficient of the media on the electromagnetic wave, which further influences the propagation speed of the electromagnetic wave in the media. The imaginary part represents the loss capacity of the media to the electromagnetic wave [32]. Penetration depth is defined as the distance that electromagnetic waves penetrate the media. The penetration depth can be written as Equation (5). According to Equation (5), the reduction coefficient α, as well as penetration depth $d_{p}$, are directly influenced by the relative permittivity of the media [33]. For concrete specimens, the proportion of moisture inside influences the reduction level of electromagnetic waves inside concrete:

(4) $$\varepsilon_{r}=\varepsilon_{r}^{\prime }-j\varepsilon_{r}^{\prime \prime }=\frac{\varepsilon }{\varepsilon_{0}}-j\frac{\delta }{\omega \varepsilon_{0}}=\varepsilon_{r}^{\prime }\left(1-j\tan\sigma \right)$$

(5) $$d_{p}=\frac{1}{\alpha }=\frac{1}{\pi }\frac{\varepsilon_{r}^{\prime }}{\varepsilon_{r}^{\prime \prime }f}\sqrt{\frac{1}{\varepsilon_{r}^{\prime }\times \varepsilon_{0}\times \mu_{0}}}$$

where $\varepsilon_{r}$ is the relative complex permittivity of media; $\varepsilon_{0}$ is the relative permittivity of a vacuum, which is 8.854 × 10^−12^ F/m; ε is the relative permittivity of the media (F/m); σ is the conductivity of media (S/m); ω is the angular frequency of the electromagnetic wave; $\varepsilon_{r}^{\prime }$ and $\varepsilon_{r}^{\prime \prime }$ are the real and imaginary parts of relative permittivity, respectively; $j=\sqrt{-1}$; $\tan\delta =\varepsilon_{r}^{\prime \prime }/\varepsilon_{r}^{\prime }$; α is reduction coefficient; $\mu_{0}$ is the magnetic permeability of vacuum, which is 4π × 10^−7^ H/m; and *f* is the frequency of electromagnetic wave.

### 2.3. Influence of Relative Permittivity on Electromagnetic Response

The transmission of electromagnetic waves inside concrete obeys the Maxwell equations, which is shown in Equations (6)–(9). Equations (10) and (11) present the definitions of permeability and permittivity, respectively, which are common constitutive forms [18,26,27]. Combining the equations mentioned above, the magnetic induction intensity is not only influenced by permeability but also permittivity, which indicates the reduction level of the electromagnetic response:

(6) $$\nabla \times E=-\frac{\partial B}{\partial t}$$

(7) $$\nabla \times H=J+\frac{\partial D}{\partial t}$$

(8) ∇×B=0

(9) ∇×D=ρ

(10) D=εE

(11) B=μH

where ρ is charge density (C/m^3^), J is current density (A/m^2^), E is the intensity of the electromagnetic wave (V/m), D is the electric displacement (C/m^2^), B is magnetic induction intensity or magnetic flux density (T), H is the magnetic field intensity (A/m), ε is the permittivity, and μ is the permeability of the media. For instance, the permittivity of an electromagnetic wave in a vacuum is 8.85 × 10^−12^ F/m and the permeability in vacuum is 4π × 10^−7^ H/m.

**Figure 1:**
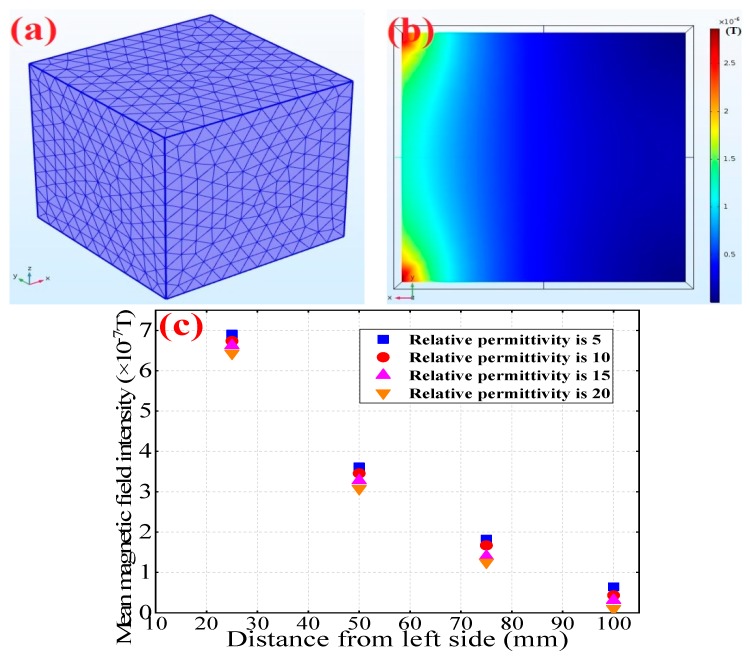
Simulation model establishment and analysis: (**a**) grid division, (**b**) magnetic field intensity distribution, (**c**) the relationship between magnetic field intensity and distance from the left side of the concrete specimen.

To further examine this phenomenon, computer software was used to simulate electromagnetic responses between two sides of concrete specimens with different relative permittivity. In the simulation process, a concrete specimen model (100 mm × 100 mm × 100 mm) was established with ordinary type meshing (Figure 1a) and a constant magnetic field (1 A/m on the *x*-axis) was applied on one side of specimen. The color cloud map shows the distribution of the magnetic flux density in the concrete cross-section, which indicates the magnetic response reduction inside concrete (Figure 1b). The relative permittivity ranged from 5 to 20 due to various moisture contents, which are reported in the literature. Figure 1b shows that no magnetic field intensity exists at the right end of concrete specimen. Figure 1c shows that the magnetic intensity contribution decreased as the distance from left side increased, which is consistent with the findings of a previous study: concrete with higher relative permittivity contributes to a higher electromagnetic wave reduction coefficient. Hence, this variation in electromagnetic response can be detected by Hall effect sensors, which can be further monitored by EMMA.

## 3. Experiments

### 3.1. Homemade Apparatus

**Figure 2:**
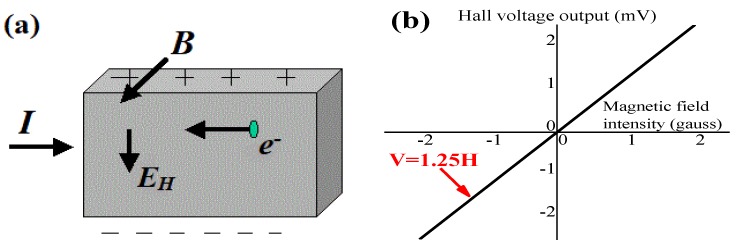
(**a**) Hall effect schematic diagram and (**b**) linear output of Hall effect sensors.

The Hall effect is a kind of electromagnetic effect that was discovered by E.H. Hall when studying the conductive mechanism of metal in 1879. When current *I* passes through the conductor perpendicular to the external magnetic field *B*, the carrier deflects and an additional electric field is generated perpendicular to the direction of the current and the magnetic field, thus generating an electric potential difference at both ends of the conductor, as shown in Figure 2a. This electric potential difference is also known as the Hall voltage difference *E_H_*. The Hall effect sensor is a kind of reliable sensor based on the Hall effect that can reflect the variation in magnetic field using linear Hall effect voltage, as shown in Figure 2b. Hall’s output voltage is divided by the midpoint voltage. Hence, the midpoint voltage (the voltage without the magnetic field) should be subtracted and the absolute value taken, and then the magnitude of the scalar magnetic field is obtained. In other words, the magnetic field intensity and the calibrated Hall voltage are basically proportional.

**Figure 3:**
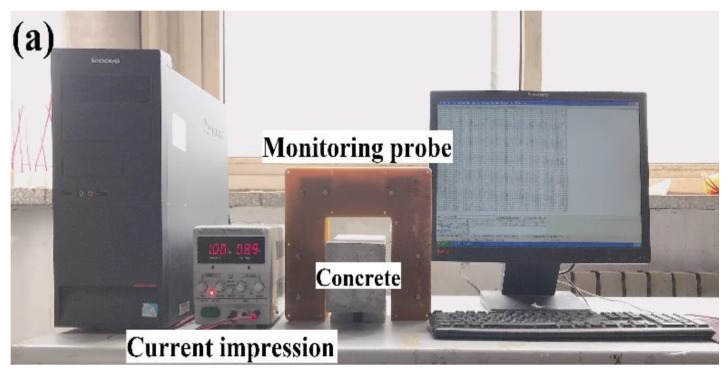

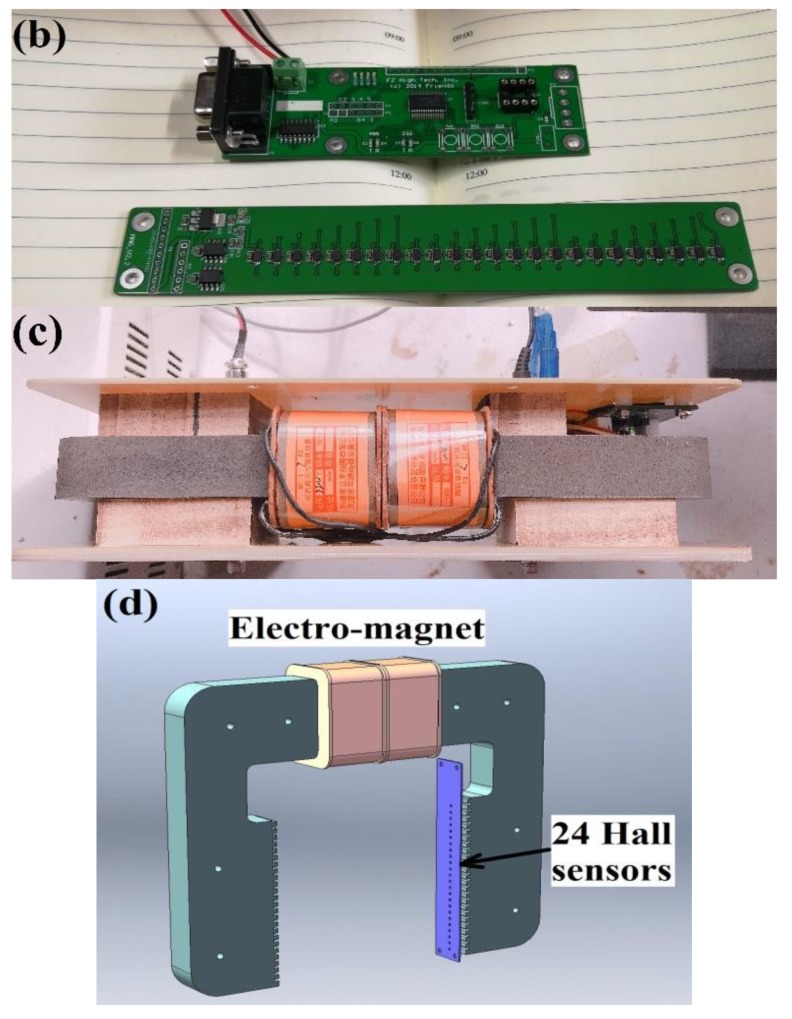
Electromagnetic monitoring apparatus (EMMA) diagram: (**a**) front view, (**b**) linear arrangement of 24 Hall effect sensors, (**c**) electromagnet (top surface), and (**d**) schematic diagram of monitoring probe.

**Figure 4:**
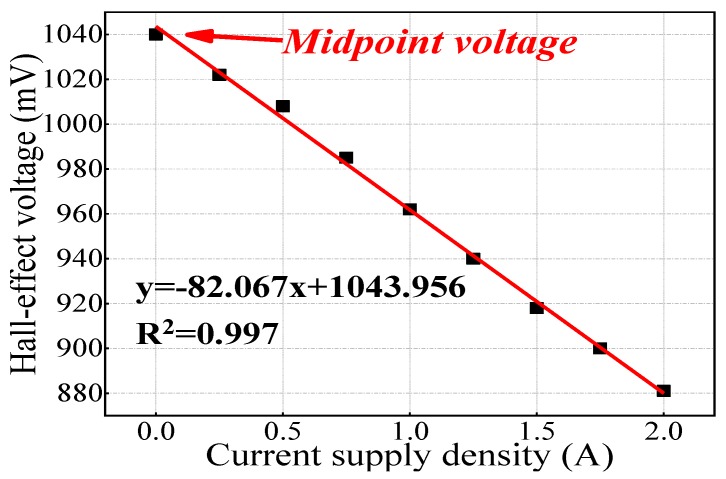
Calibration process of homemade apparatus.

By using Hall effect sensors and generating a stable magnetic field, concrete with different relative permeabilities will have various magnetic intensities, which can be acquired by EMMA and presented in the form of Hall effect signals. A–1391-type Hall effect sensors were used on this EMMA version, whose sensitivity approaches 1.25 mV/G, as is shown in Figure 2b. Figure 3a shows the diagram of EMMA and Figure 3b shows how 24 Hall sensors were linearly arranged, containing 24 channels to collect data. An electromagnet was placed on the top of the monitoring probe to provide a stable magnetic field for reinforced concrete specimens with an external direct current supply (Figure 3c). Figure 3d shows a schematic diagram of the monitoring probe. The magnetic field generated by the electromagnet is applied to one side of the concrete samples by the iron monitoring probe and the magnetic field that reaches the other side of the sample is measured by the 24 Hall sensors. The signals received by Hall sensors were transformed to computer language and shown on the serial port assistant. The monitoring probe has an embedded A/D converter and an indication light. The inner length of the monitoring probe is 120 mm, so it is able to scan cross-sections of cubic concrete specimens whose side length is 100 mm. In accordance with electromagnetic theory, the magnetic field intensity inside the monitoring probe is linearly related with the direct current supply provided by an external supply device. A proportional relationship exists between supply current density and mean Hall effect voltage variation of 24 Hall sensors (Figure 4). It is clearly shown in Figure 4 that Hall effect voltage shows the reverse tendency with supply current density. It is worth noting that the midpoint voltage (Hall signals received by Hall elements when no current impressed) is approximately 1040 mV. Moreover, the Hall signal’s variable quantity is defined as the absolute value of difference between monitoring data and midpoint voltage (1040 mV). The self-calibration process was previously reported [18].

**Figure 5:**
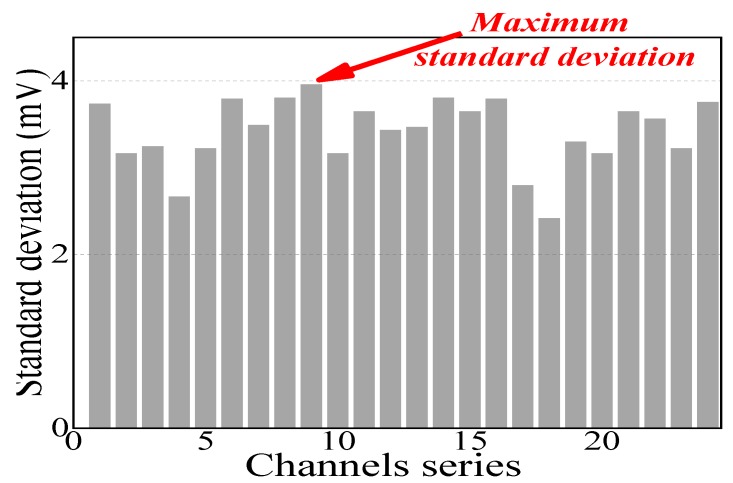
Standard deviation of 24 channels.

Different Hall effect sensors have unique average signal profiles. Hence, the standard deviation of every single Hall element was obtained when the current supply density was 1 A with continuous data collection. Figure 5 shows the standard deviation of the 24 Hall element channels calculated using Equation (12) and the mean value that can be recognized as the current supply is 1 A, which presented autologous fluctuation triggered by the geomagnetic field and the transition coefficient of the A/D converter when no corrosion was present. The original collected data were obtained from the A/D converter whose unit is counts, then multiplying by a coefficient of 0.133 mV to transform into Hall effect potentials. Figure 5 shows that the maximum standard deviation of the 24 test channels was 3.9 mV; hence, ±4 mV was regarded as a boundary condition to evaluate the electromagnetic response variation excluding the impact of the self-fluctuation of monitoring data:

(12) $$\delta_{a_{ij}}=\sqrt{\frac{1}{n}\sum_{j=1}^{j=n}{\left(a_{ij}-\frac{1}{n}\sum_{j=1}^{j=n}a_{ij}\right)}^{2}\;}\;(1\;\leq i\;\leq 24,\;j\;\geq 1)$$

where $a_{ij}$ was defined as an element in the signal matrix shown in the serial assistant software, which means that the value was recorded by a Hall effect sensor whose sensor series is *i*; meanwhile, *j* represents the *j*th data recorded by the *i* sensor. *n* represents the collection times.

### 3.2. Materials

**Figure 6:**
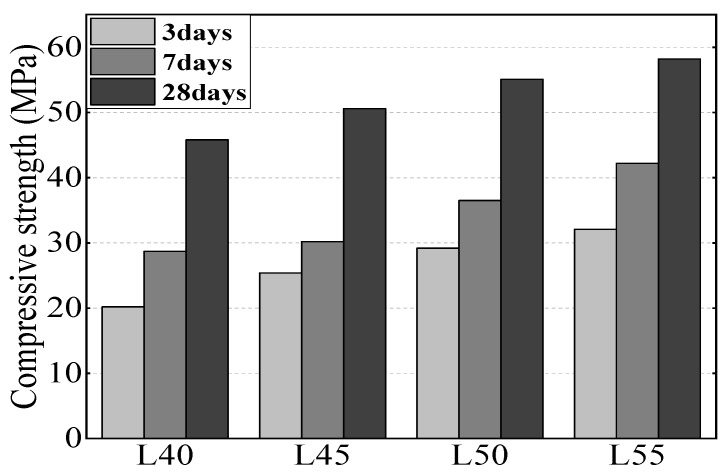
Compressive strength of concrete at different curing ages.

P.I.42.5 Portland cement, in accordance with Chinese standard GB175-2007 (ordinary Portland cement with Japanese Standard JIS R5210 or type 42.5 Portland cement with English Standard EN197 equivalent) from Shanshui Co., Ltd. (Qingdao, China), was used in this study. Class I fly ash (per Chinese standard GB1596–2005) from Huadian Corporation, and S95 GGBS (Chinese standard GB/T18046-2008) from Zhongkuang Corporation were employed to replace Portland cement. The chemical composition and physical characteristics of cement, fly ash, and GGBS are shown in Table 1 and Table 2, respectively. The mix proportions of the concretes are shown in Table 3 and the compressive strength of concrete at different curing times is shown in Figure 6. The composition of seawater used in the water absorption test is shown in Table 4. Because too large an aggregate is not conducive to improving the compactness of concrete, and it may also increase the chloride ion diffusion coefficient, crushed granite with a maximum size of 25 mm was used as the coarse aggregate and river sand with fineness modulus of 2.6 was used. Polycarboxylic acid super plasticizer from Sobute New Materials Co., Ltd. (Nanjing, China) was used and its proportion was adjusted to maintain the slump of fresh concrete within the range of 140 mm to 180 mm. Based on trials and durability tests, a high mineral content admixture including GGBS and fly ash could improve the workability of fresh concrete and chloride bound capacity; a 40–50% mineral admixture replacement was used to prepare the concrete.

### 3.3. Specimen Fabrication

**Figure 7:**
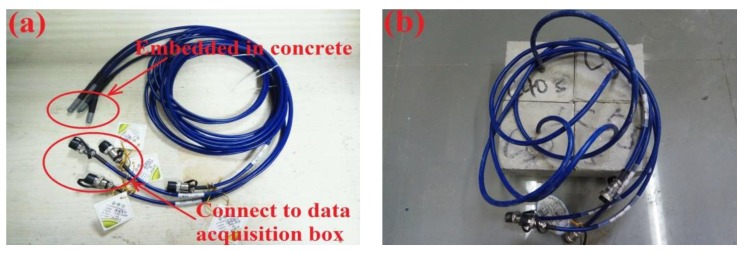
Temperature and relative humidity sensor: (**a**) monitoring probe and (**b**) probe embedded in concrete.

Four groups of 100 mm × 100 mm × 100 mm specimens for a total of 76 cubes were fabricated. After casting, these molds were rotated slowly until pastes were totally hardened. All the specimens were placed in the mold, then removed after 48 h, and then cured at 20 ± 3 °C and 95% relative humidity for 28 days. To monitor the relative humidity inside the concrete specimens, a temperature and relative humidity sensor produced by Sanzhi Electronic Technology Co., Ltd. (Changsha, China) was used (Figure 7). Four relative temperature and humidity sensors were embedded in four concrete specimens with four different concrete mix proportions, as shown in Figure 7b. Table 5 shows the relevant parameters of this sensor. The prepared concrete specimens for relative humidity, carbonation, and water amount experiment were labeled groups A, B, and C, respectively, and each group included all four different w/b ratio concretes.

### 3.4. Experimental Method

Temperature directly affects the relative humidity (RH) inside concrete [34,35,36,37]; hence, for Group A, temperature was controlled as constant in this experiment. Four concrete specimens with temperature and RH sensors were placed in a room whose indoor temperature was kept steady. After de-molding and curing, the RH inside concrete was 100% and the hydration process was still ongoing. Then, these four specimens were placed in the monitoring probe shown in Figure 3a to record the signals from the 24 Hall effect sensors. The data recorded by EMMA were the original data output from the A/D converter; therefore, Hall voltage was converted based on the transition coefficient of the A/D converter. In this experiment, the relationship between Hall voltage and relative humidity inside concrete was established.

As for Group B, concrete specimens were exposed to a carbonation tank with 75% RH and a 20% CO_2_ concentration. Three specimens were removed from the tank and placed into the monitoring probe to evaluate Hall signals after 3 days, 7 days, 14 days, 28 days, and 56 days of carbonation. Then, the specimens were split and sprayed with phenolphthalein solution to measure average carbonation depth. The relationship between Hall voltage and carbonation depth was established.

All the specimens of Group C after curing were placed in a drying oven at 60 °C until the specimens reached a constant weight (so-called dry concrete). After, all the specimens of Group C were covered with tinfoil paper so that the seawater could only migrate inside the concrete from one direction. All specimens were placed on the bottom of a tank filled with sea water in an experimental room at constant RH (50% ± 10%) and temperature (20 ± 0.5 °C), then the seawater adsorption experiment was conducted. The mass variation of the concrete specimens was evaluated after 0.5 h, 1 h, 2 h, 4 h, 8 h, 12 h, and 24 h; Hall signals were simultaneously monitored. Different from internal relative humidity inside the concrete, the moisture absorption experiment was conducted allowing seawater to migrate from one end of the concrete specimen to the other and average weight was measured. As previously, the relationship between Hall voltage and the amount of water absorption was established.

## 4. Results and Discussion

### 4.1. Relative Humidity Effect

**Figure 8:**
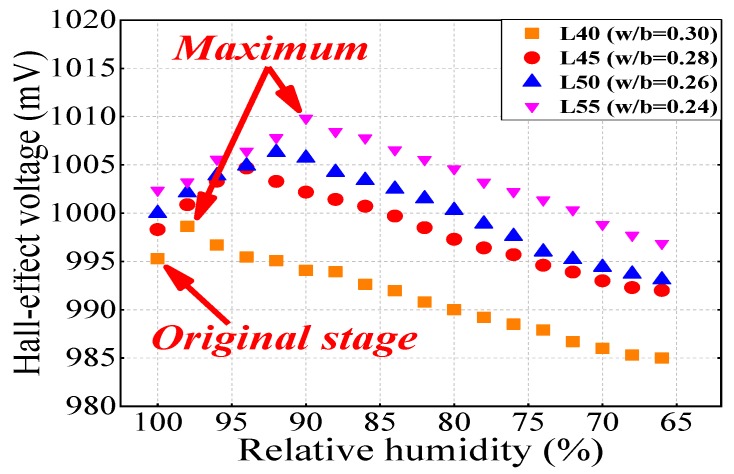
The relationship between Hall effect voltage and relative humidity.

Four concrete specimens were embedded with temperature and RH sensors to monitor relative humidity, and then Hall effect voltage signals were detected using EMMA. Figure 7 shows the relationship between Hall effect voltage and relative humidity. Like the maximum chloride ions phenomenon at the surface of concrete, the Hall signals first increased to the top and then decreased at a certain RH. The RH value for the maximum Hall voltage also showed a decreasing trend as the w/b ratio decreased, and also, the maximum Hall voltage increases as w/b ratio decreases. The original state in Figure 8 indicates that the relative permeability of concrete significantly affects the moisture content inside concrete, although RH was 100% for both. A longer time was required for L55 to reach the maximum RH of approximately 90%, which was approximately 98% for L40. Previous studies [38,39,40] also reported a similar tendency of concrete with different w/b ratios in homogeneous magnetic fields due to different microstructural characteristics such as the surface/volume (S/V) ratio of the pores. Two basic aspects of concrete, drying shrinkage and moisture migration, initiate from the concrete surface; then, the internal concrete characteristics can be explained based on these aspects, which have been widely reported in the literature [22,41].

### 4.2. Carbonation Exposure Effect

**Figure 9:**
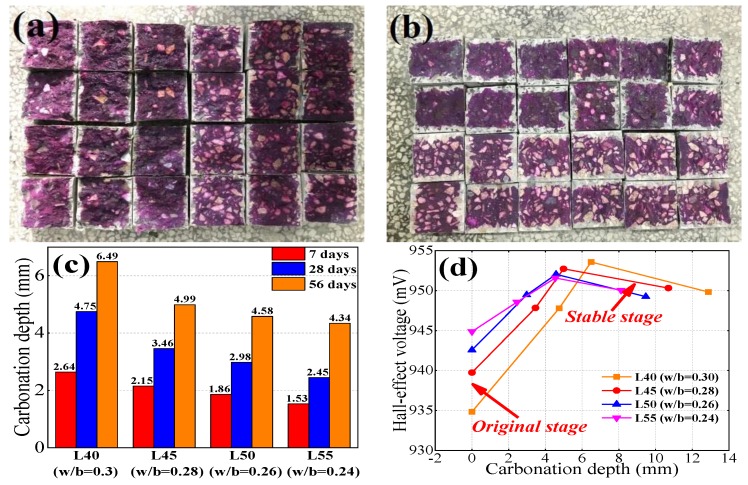
Carbonation test after (**a**) 14 days and (**b**) 56 days of exposure. (**c**) Carbonation depth and (**d**) the relationship between Hall voltage and carbonation depth.

With increasing carbonation exposure time, the un-discolored area increases gradually, which indicates the increasing average carbonation depth (Figure 9a,b). Figure 9d shows the relationship between Hall effect voltage signals and average carbonation depth. The Hall signals increased during the initial stage of carbonation, and then stabilized as carbonation depth increased. According to Figure 9d, the electromagnetic signals did not significantly vary when the carbonation depth was more than 4 mm, which means that calibration is unnecessary when carbonation depth is more than 4 mm. Concrete with a higher w/b ratio is more vulnerable to carbonation, as confirmed by the average carbonation depth in Figure 9c. Figure 9d shows that the Hall signal variation increased with increasing w/b ratio; in other words, concrete with a higher w/b ratio has more Hall signal variation and much less of a need to be calibrated. Kim [23] highlighted that CaCO_3_ fills the pores and reduces the relative permeability during carbonation process, which contributes to the positive tendency of the Hall effect voltage as carbonation depth increases.

### 4.3. Water Content Absorption

**Figure 10:**
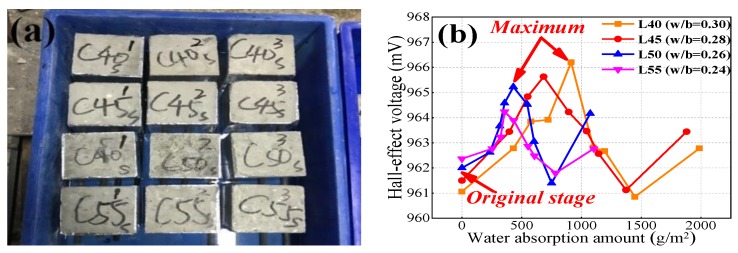
Water absorption test: (**a**) experimental diagram and (**b**) the relationship between Hall effect voltage and water absorption amount.

After Group C specimens were all placed in the tank with seawater in the bottom, they were removed to evaluate the amount of absorbed water at 0.5 h, 1 h, 2 h, 4 h, 8 h, 12 h, and 24 h (Figure 10a). Figure 10b shows the relationship between Hall voltage and water absorption amount. Figure 10b shows that Hall signals initially increased with increasing water absorption amount, and the trend later reversed, then increasing from the minimum. Figure 10b also shows that the maximum value increased as *the* w/b ratio decreased; in other words, the variation in the Hall signals decreased with increasing w/b ratio. Hence, Hall signals received from concrete specimens after carbonation exposure need to be calibrated. In terms of other influential factors, L55 had the lowest Hall signal variation due to having the lowest w/b ratio.

### 4.4. Calibration System Establishment

According to the experimental data, a calibration system was established. Here, a w/b ratio of 0.3 (standard compressive strength of 40 MPa after curing for 28 days) was regarded as one. The RH curve increased initially and then decreased after climbing to the maximum point. Considering this, the linear function was used to create the curve, and the scope of fitting linear formula was calculated and considered as a correction factor. Table 6 shows the calibration procedure for RH. The entire curve was divided into two linear straight lines and the boundary point was defined with relation to 98% (critical point of L40). For the carbonation process, as mentioned above, no calibration is required when the average carbonation depth is more than 4 mm (stable stage). As stated previously, Hall signals during carbonation procedure were fitted using linear fitness, as shown in Table 7.

**Figure 11:**
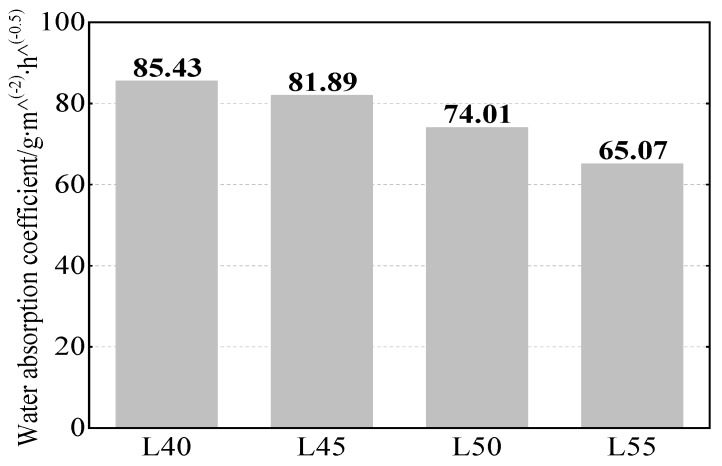
Water absorption coefficient of different w/b ratio concretes.

Calibrating Hall signals for the water absorption experiment was relatively complicated, as the water absorption coefficient needs to be calculated first. The water absorption coefficient is defined in Equation (13):

(13) $$\Delta W/A=\Delta \sqrt{t}$$

where ΔW is the water absorption amount at time *t* (g/m^2^), *A* is the water absorption coefficient (g/m^2^h^0.5^), and *t* is absorption time (h). Figure 11 also shows the water absorption coefficient for the four w/b ratios, which indicates that the water absorption coefficient decreased as the w/b ratio decreased. Table 8 shows the calibration procedure for Hall signals for the water absorption experiment.

In this calibration procedure, only linear fitting formulas were considered when establishing this system. The calibration procedure is only valid for this kind of homemade apparatus. Notably, only moisture content inside the concrete was considered as the influencing factor in this study. However, temperature, electromagnetic wave frequency, and aggregate type can also impact the electromagnetic response, which will require investigation in the future.

## 5. Conclusions

In this study, a homemade apparatus was used to evaluate the electromagnetic response of concrete specimens with different moisture contents and affected by carbonation. Our main conclusions are as follows:

1. The moisture content inside the concrete influences the electromagnetic response of concrete, which includes variation in relative humidity, carbonation exposure, and water absorption. Relative humidity influences the electromagnetic response the most and the water absorption amount has the least impact.
2. The reduction coefficient is significantly influenced by the relative permittivity of the media, which contributes to various electromagnetic reduction levels inside the concrete. Concrete with a higher moisture content has a higher electromagnetic reduction coefficient.
3. A calibration system was established to provide a signal amendment procedure for the electromagnetic monitoring apparatus in a specific area to improve the accuracy of monitoring signals. All the values of the correction factors were given.

## Figures and Tables

**Table 1:** Chemical composites of binder materials (wt. %).

Constituent	SiO_2_	Al_2_O_3_	Fe_2_O_3_	CaO	MgO	Na_2_O	K_2_O	SO_3_	P_2_O_5_
Cement	19.80	4.18	3.63	62.03	4.48	0.96	0.48	1.88	0.10
GGBS	32.58	13.27	1.34	41.06	5.62	0.45	0.54	2.68	0.04
Fly ash	56.90	13.70	4.40	1.96	0.32	0.17	1.10	0.57	0.10

**Table 2:** Physical characteristics of cement, fly ash, and GGBS.

	Density (kg/m^3^)	Fineness	Particle Size (3–65 µm, %)
Cement	3050	360 m^2^/kg (specific surface area)	82.93
GGBS	2810	379 m^2^/kg (specific surface area)	75.45
Fly ash	2050	10.5% remained (45 μm griddle)	78.65

**Table 3:** Mix proportions of concrete (kg/m^3^). w/b = water/binder ratio.

	Binder	Cement	GGBS	Fly Ash	Sand	Granite Stone	Super Plasticizer	Water	w/b
L40	346	173	86	86	780	1169	4.5	104	0.3
L45	384	192	96	96	761	1142	5.0	108	0.28
L50	435	217	109	109	738	1108	5.7	113	0.26
L55	474	237	118	118	721	1081	6.2	119	0.24

**Table 4:** Composition of seawater (mg/L).

$NO_{3}^{-}$	${\mathrm{HCO}}_{3}^{-}$	${\mathrm{SO}}_{4}^{2-}$	${\mathrm{Cl}}^{-}$	${\mathrm{NH}}_{4}^{+}$	${\mathrm{Ca}}^{2+}$	${\mathrm{Mg}}^{2+}$	pH
12.76	161.29	2176.12	17,533.33	0.04	407.83	1177.38	6.98

**Table 5:** Relevant parameters of temperature and relative humidity sensors.

Accuracy of Temperature (°C)	Accuracy of Relative Humidity (RH)	Temperature Range (°C)	RH Range	Working Voltage (V)	Channels
0.1	±2%	–40 to 80	0–100%	5	4/8/16

**Table 6:** RH calibration procedure.

w/b	Fitting Formula (Ascending Stage)	Fitting Formula (Descending Stage)	Critical Point (Ratio to RH 98%)	Correction Factor (Descending Stage)	Correction Factor (Descending Stage)
0.30	y_A_ = –1.671x + 1162.286 (*R^2^* = 0.999)	y_D_ = 0.401x + 958.067 (*R^2^* = 0.996)	98% (1)	1 (standard)	1 (standard)
0.28	y_A_ = –1.078x + 1106.278 (*R^2^* = 0.985)	y_D_ = 0.463x + 960.697 (*R^2^* = 0.994)	96% (0.98)	1.55	1.15
0.26	y_A_ = –0.768x + 1007.194 (*R^2^* = 0.976)	y_D_ = 0.544x + 956.493 (*R^2^* = 0.993)	94% (0.96)	2.17	1.36
0.24	y_A_ = –0.740x + 1076.206(*R^2^* = 0.980)	y_D_ = 0.580x + 961.198 (*R^2^* = 0.998)	90% (0.92)	2.26	1.45

**Table 7:** Carbonation depth calibration procedure.

w/b	Fitness Formula	Correction Factor
0.30	y_C_ = 2.855x + 934.704 (*R^2^* = 0.998)	1 (standard)
0.28	y_C_ = 2.559x + 939.563 (*R^2^* = 0.994)	1.12
0.26	y_C_ = 2.101x + 942.732 (*R^2^* = 0.993)	1.36
0.24	y_C_ = 1.477x + 944.901 (*R^2^* = 0.999)	1.93

**Table 8:** Calibration procedure for the water absorption experiment.

w/b	Water Absorption Coefficient (g/m^2^h^0.5^)	Correction Factor
0.30	85.43	1
0.28	81.89	1.04
0.26	74.01	1.15
0.24	65.07	1.31
